# Exploring the Effect of a MnO_2_ Coating on the Electrochemical Performance of a Li_1.2_Mn_0.54_Ni_0.13_Co_0.13_O_2_ Cathode Material

**DOI:** 10.3390/mi12111410

**Published:** 2021-11-17

**Authors:** Zhong Li, Peiyue Yang, Zhongxiang Zheng, Qiyun Pan, Yisi Liu, Yao Li, Jinnan Xuan

**Affiliations:** Institute for Advanced Materials, Hubei Normal University, Huangshi 435002, China; ypy0830@163.com (P.Y.); zzx18370065140@163.com (Z.Z.); qypan@hbnu.edu.cn (Q.P.); yliu88@hbnu.edu.cn (Y.L.); yaoli@hbnu.edu.cn (Y.L.); jnxuan@hbnu.edu.cn (J.X.)

**Keywords:** lithium-rich cathode material, surface modification, manganese dioxide, electrochemical performance

## Abstract

The effect of electrochemically active MnO_2_ as a coating material on the electrochemical properties of a Li_1.2_Mn_0.54_Ni_0.13_Co_0.13_O_2_ (LTMO) cathode material is explored in this article. The structural analysis indicated that the layered structure of the LTMO was unchanged after the modification with MnO_2_. The morphology inspection demonstrated that the rod-like LTMO particles were encapsulated by a compact coating layer. The MnO_2_ layer was able to hinder the electrolyte solution from corroding the LTMO particles and optimized the formation of a solid electrolyte interface (SEI). Meanwhile, lithium ions were reversibly inserted into and extracted from MnO_2_, which afforded an additional capacity. Compared with the bare LTMO, the MnO_2_-coated sample exhibited enhanced electrochemical performance. After the MnO_2_ coating, the first discharge capacity rose from 224.2 to 239.1 mAh/g, and the initial irreversible capacity loss declined from 78.2 to 46.0 mAh/g. Meanwhile, the cyclic retention climbed up to 88.2% after 100 cycles at 0.5 C, which was more competitive than that of the bare LTMO with a value of 71.1%. When discharging at a high current density of 2 C, the capacity increased from 100.5 to 136.9 mAh/g after the modification. These investigations may be conducive to the practical application of LTMO in prospective automotive Li-ion batteries.

## 1. Introduction

Developing high-energy-density secondary batteries to appease the increasing consumption of electronic apparatuses and electric vehicles is the responsibility of battery researchers [1,2]. In the last two decades, Li-rich Mn-based solid-solution cathode materials of *n*Li_2_MnO_3_**·**(1−*n*)LiTMO_2_ (0 < *n* < 1, TM = Mn, Ni, Co, Mn_0.5_Ni_0.5_, Mn_0.333_Ni_0.333_Co_0.333_, etc.) with a layered structure have drawn much attention, which has been ascribed to their outstanding specific capacity [3,4,5]. The *n*Li_2_MnO_3_**·**(1−*n*)LiTMO_2_ materials are made up of a monoclinic Li_2_MnO_3_ component (C2/m) and a layered LiTMO_2_ component (R-3m) [6,7]. Usually, the two-component notation *n*Li_2_MnO_3_**·**(1−*n*)LiTMO_2_ can be denoted as a layered type of Li_1+*n*_TM_1−*n*_O_2_ [8]. The cutoff voltage of Li_1+_*_n_*TM_1−_*_n_*O_2_ is high—up to 4.6–4.8 V—because the Li_2_MnO_3_ component must undergo an activation over 4.5 V to generate an extra reversible capacity, enabling Li_1+_*_n_*TM_1−_*_n_*O_2_ to achieve a much higher specific capacity than that of the LiTMO_2_ component [4,9].

As one of the most representative types of Li_1+_*_n_*TM_1−_*_n_*O_2_, Li_1.2_Mn_0.54_Ni_0.13_Co_0.13_O_2_ (LTMO, or denoted as 0.5Li_2_MnO_3_·0.5LiMn_0.333_Ni_0.333_Co_0.333_O_2_) can release a competitive capacity of 220–280 mAh/g at 2.0–4.8 V [10,11,12], and it is considered to be one of the preferred cathode materials for prospective automotive Li-ion batteries. However, LTMO still suffers from several shortcomings that limit its practical application [8,9,10,11]. During the first charge, the movement of transition metal ions eliminates oxide ion vacancies generated by the deintercalation of lithium as Li_2_O in the Li_2_MnO_3_ over 4.5 V, which gives rise to a decrease in lithium-ion sites for Li^+^ to be reinserted during the first discharge [3,13]. The reduced insertion of lithium ions, along with the growth of a thick solid electrolyte interface (SEI) layer caused by the side reaction between the LTMO particles and electrolyte, brings about a large initial irreversible capacity loss for the LTMO [4,6]. Meanwhile, the continuous corrosion of the electrolyte on the LTMO surface at a high cut-off voltage of 4.8 V severely deteriorates the cyclic performance [9,14]. Moreover, the LTMO demonstrates an unsatisfied rate capability, which is ascribed to the poor conductivity for the electrons and lithium ions of the Li_2_MnO_3_ component [7,11]. 

Currently, many approaches have been implemented to upgrade the electrochemical properties of LTMO, including morphology optimization [15,16,17], ion doping [18,19,20,21,22], surface coating [10,11,13,14,23,24,25,26,27,28,29,30,31,32,33,34], and special treatment [35,36,37,38]. Among the above-mentioned strategies, modification of the surface of LTMO particles with inert metal oxides has been testified to remarkably enhance the cyclic performance, such as with ZrO_2_ [10], TiO_2_ [25], Al_2_O_3_ [23,26], MgO [27], ZnO [28], Pr_6_O_11_ [29], and Er_2_O_3_ [30]. The above chemically stable metal oxides not only stabilize the interface of LTMO particles, but also act as an effective barrier in order to inhibit the etching by the electrolyte [10,23,25,26,27,28,29,30]. Nevertheless, owing to the electrochemical inertness properties of these metal oxides, the coating layer will reduce the initial specific capacity of LTMO to some extent [10,25,26,27,28,29,30].

MnO_2_ is a well-known electrochemically active oxide that has been widely applied as an active material for the research fields of supercapacitors [39], electrocatalysis [40], and lithium-ion batteries [41]. Recently, the function of MnO_2_ has extended to become a surface modification material on cathode materials, such as LiMn_2_O_4_ [42], Li_3_V_2_(PO_4_)_3_ [43], and LiMn_0.333_Ni_0.333_Co_0.333_O_2_ [44], in order to obtain a better electrochemical performance. Firstly, the stable MnO_2_ protective layer can hinder the electrolyte from corroding the cathode particles and can optimize the formation of the SEI layer [42,43,44]. Furthermore, lithium ions can be inserted into and extracted from the electrochemically active MnO_2_ under 3 V, which affords an additional capacity and benefits the Li^+^ transfer from the interface of the cathode particle to the electrolyte [44].

Inspired by such research, this article explores the impacts of MnO_2_ as a surface modification material on LTMO particles. The structures and morphologies of LTMO before and after MnO_2_ modification are thoroughly examined. In addition, the electrochemical properties, such as the specific capacity, initial irreversible capacity loss, cyclic stability, rate capability, and electrochemical impedance spectroscopy, are systematically investigated and discussed.

## 2. Materials and Methods

### 2.1. Preparation of Bare LTMO 

The bare LTMO cathode material was synthesized as follows: Firstly, stoichiometric amounts of manganese sulfate monohydrate, nickel sulfate hexahydrate, and cobalt sulfate heptahydrate were put into distilled water and stirred continuously to generate a homogeneous solution (1 mol/L). Then, 1 mol/L sodium carbonate solution and 0.3 mol/L ammonium hydroxide were slowly dropped into the above solution. After being stirred at 60 °C for 0.5 h and 30 °C for 12 h, the coprecipitated carbonate precursor was filtered and then rinsed with distilled water. After drying at 80 °C for one day, the filtered powder was thoroughly blended with lithium carbonate, then heated at 500 °C for 5 h and calcined at 900 °C for 15 h in air atmosphere to form LTMO.

### 2.2. Preparation of MnO_2_-Coated LTMO

The MnO_2_-coated LTMO was prepared as follows: Initially, the LTMO was put into the manganese sulfate solution and stirred vigorously for 0.5 h. Then, an aqueous solution of sodium carbonate was dropped slowly into the above suspension to precipitate manganese carbonate. The obtained slurry was filtered and rinsed with distilled water, subsequently dried at 80 °C for 2 days, and then heated at 400 °C for 2 h in air atmosphere to get the MnO_2_-coated LTMO material with 3 wt % MnO_2_.

### 2.3. Material Characterization 

The materials were characterized by using X-ray diffraction (XRD, D8-Advance, Bruker, Karlsruhe, Baden-Württemberg, Germany) (2*θ* degree: 10–70°, operating rate: 4°/min). The particle micro-morphologies were inspected by using field-emission scanning electron microscopy (FESEM, Sirion 200, FEI, Hillsboro, OR, USA), and the elemental distribution was probed with energy-dispersive spectroscopy (EDS, SU8010, HITACHI, Tokyo, Japan). The specific amounts of transition metal ions were determined by using inductively coupled plasma atomic emission spectrometry (ICP-AES, Optima 7000 DV, PerkinElmer, Waltham, MA, USA). The MnO_2_ coating layer on the LTMO particles was surveyed by using transmission electron microscopy (TEM, Philips CM12, Philips, Amsterdam, Noord-Holland, Netherlands). 

### 2.4. Electrochemical Evaluations

CR2016 coin cells were fabricated to evaluate the electrochemical properties. To prepare the LTMO (bare and MnO_2_-coated) electrodes, active cathode powder (85 wt %), conductive carbon (10 wt %), and polytetrafluoroethylene (PTFE) (5 wt %) were mixed and continuously rolled to obtain a thin sheet. Subsequently, the thin sheet was separated into several circular films and dried at 120 °C in a vacuum to produce the electrodes. The diameter and mass loading of the LTMO (bare and MnO_2_-coated) electrodes were 8 mm and 5~6 mg/cm^2^. The cells were fabricated with the electrodes, metallic lithium sheet, separator (Celgard 2400), and carbonate-based electrolyte (1 mol/L LiPF_6_ in the solvents consisting of ethylene carbonate and dimethyl carbonate with the volume ratio of 1:1) under an argon atmosphere. The galvanostatic charge/discharge evaluations were implemented at ambient temperature on an electrochemical test system (CT2001A, Wuhan Land) at 2.0–4.8 V with various current densities (1 C = 250 mA/g). Electrochemical impedance spectroscopy (EIS) was assessed by utilizing a CorrTest electrochemical workstation (frequency: 100,000–0.01 Hz, amplitude: 10 mV).

## 3. Results and Discussion

### 3.1. Structures of Bare and MnO_2_-Coated LTMO

**Figure 1:**
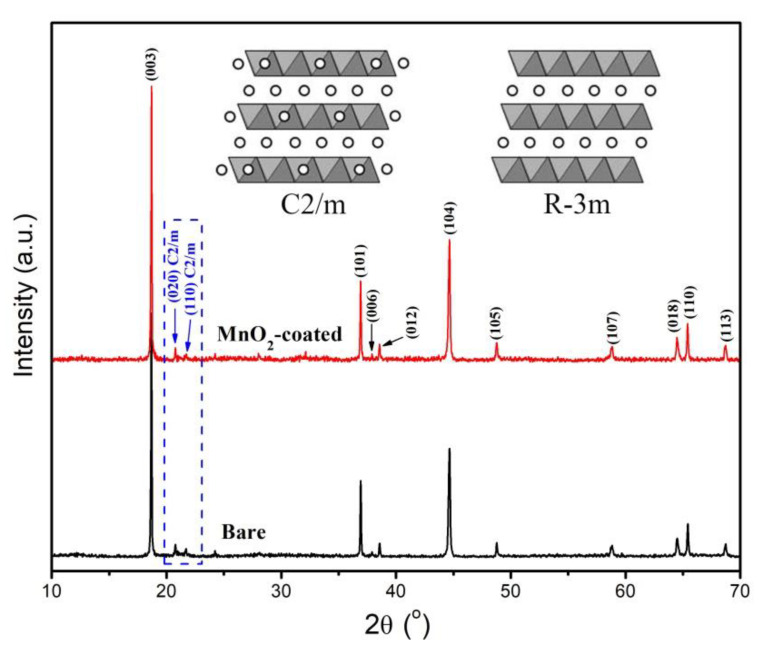
XRD results of bare and MnO_2_-coated LTMO.

The XRD results for the bare and MnO_2_-coated LTMO are exhibited in Figure 1. All of the diffraction peaks (excluding the peaks from 20° to 25°) of both samples belonged to the layered LiMn_0.333_Ni_0.333_Co_0.333_O_2_ component with a hexagonal α-NaFeO_2_ structure (R-3m) [13]. The weak peaks (020) and (110) located at the degree of 20–25° indicate the existence of the monoclinic Li_2_MnO_3_ component (C2/m) [27]. The (006)/(012) and (018)/(110) peaks displayed obvious separation in the bare and MnO_2_-coated LTMO, proving the excellence of the layered structure [15,20]. 

The lattice parameters of the bare and MnO_2_-coated LTMO from XRD patterns are presented in Table 1. Since the ion radii of Ni^+^ and Li^+^ were very close, cation mixing may have occurred in the preparation process of the materials. If the values of *c*/*a* and *I*_(003)_/*I*_(104)_ of 4.96 and 1.2 cannot be achieved, this implies that partial cation mixing exists in a material [16]. Notably, the *c*/*a* and *I*_(003)_/*I*_(104)_ of both materials demonstrated values higher than 4.99 and 2.2, respectively, indicating the low degree of cation mixing for the bare and MnO_2_-coated LTMO. Furthermore, no distinct XRD peaks were indexed to MnO_2_ in the MnO_2_-coated LTMO, which may have been caused by the low amount and poor crystallinity of MnO_2_ [42,43,44]. The XRD results illustrate that the MnO_2_ coating did not damage the lattice structure of the LTMO.

### 3.2. Microscopic Morphologies of Bare and MnO_2_-Coated LTMO

**Figure 2:**
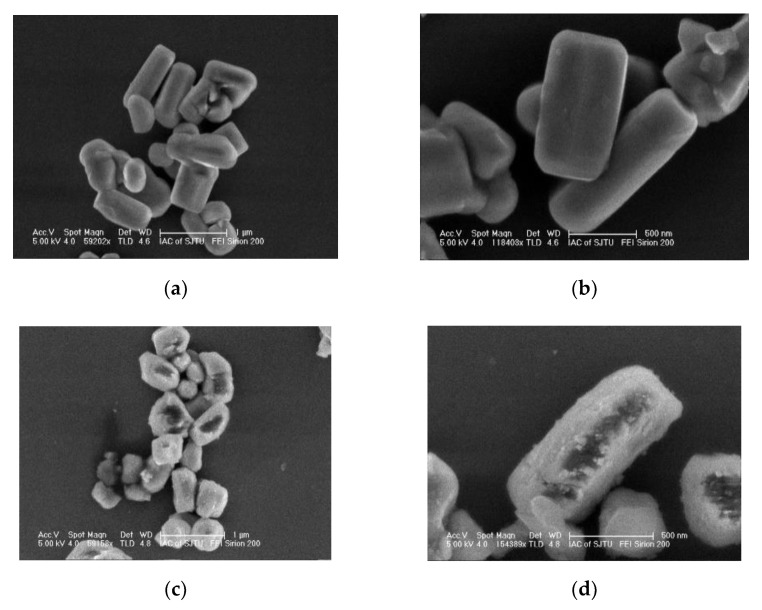
FESEM results for the (**a**,**b**) bare and (**c**,**d**) MnO_2_-coated LTMO particles.

The field-emission scanning electron microscopy (FESEM) images of the LTMO particles before and after the MnO_2_ modification are shown in Figure 2 with different magnifications. The images in Figure 2a,c show that both samples comprised well-crystallized particles, and the particles presented a rod-like morphology with lengths in the range of 300–1000 nm. The bare LTMO particles all displayed clean and smooth surfaces at high magnification (Figure 2b). In contrast, the MnO_2_-coated LTMO particles exhibited ambiguous and rough surfaces, which were ascribed to the coarse coating layer (Figure 2d).

**Figure 3:**
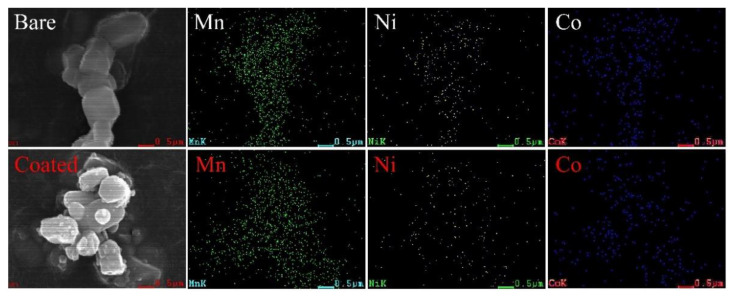
The elemental distributions of Mn, Ni, and Co in the bare and MnO_2_-coated LTMO particles.

The elemental distributions of Mn, Ni, and Co in the bare and MnO_2_-coated LTMO particles are shown in Figure 3. For both samples, the transition metal elements were uniformly dispersed in the particles. Moreover, the ICP-AES test was applied to examine the elemental composition of transition metal ions in the two samples, and the measured values are presented in Table 2. The atomic ratio in the bare LTMO was almost identical to the theoretical stoichiometric ratio of Mn, Ni, and Co (0.54:0.13:0.13). It should be noted that the content of the Mn element in the MnO_2_-coated LTMO was 3.327%, which was higher than in the bare simple. This result originated from the added MnO_2_ coating layer and was very close to the designed amount of the coated Mn element (3 wt %) in this article.

**Figure 4:**
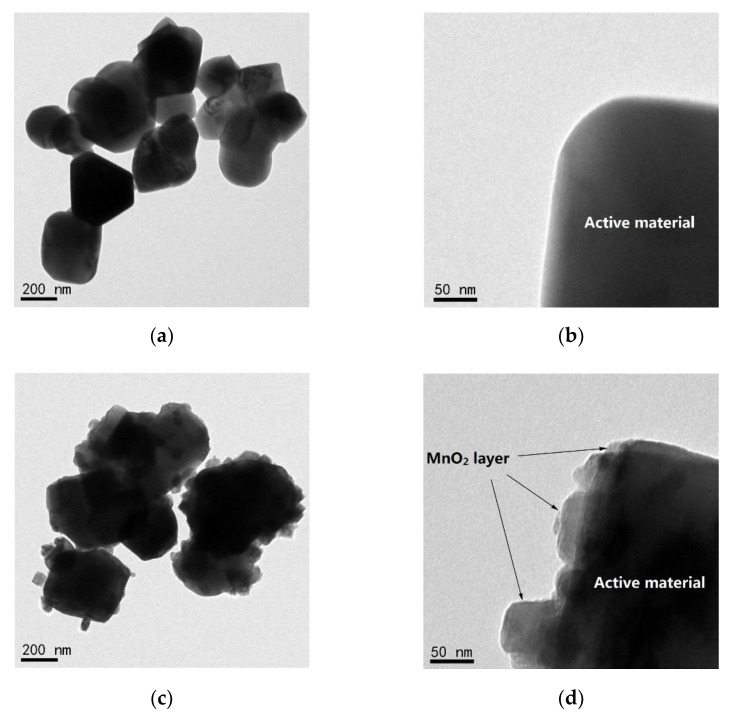
TEM images of the (**a**,**b**) bare and (**c**,**d**) MnO_2_-coated LTMO particles.

Figure 4 demonstrates TEM images of the bare and MnO_2_-coated LTMO particles. Compared to the smooth surface appearing in the bare sample at high magnification (Figure 4b), it can be seen that the MnO_2_-coated LTMO in Figure 4d was coated with a compact and distinguishable layer with a thickness from 10 to 50 nm. The compact coated layer was able to encapsulate the LTMO particles to constrain the side reaction and erosion caused by the electrolyte [42,43,44].

### 3.3. The Electrochemical Characteristics of Bare and MnO_2_-Coated LTMO at 0.05 C

**Figure 5:**
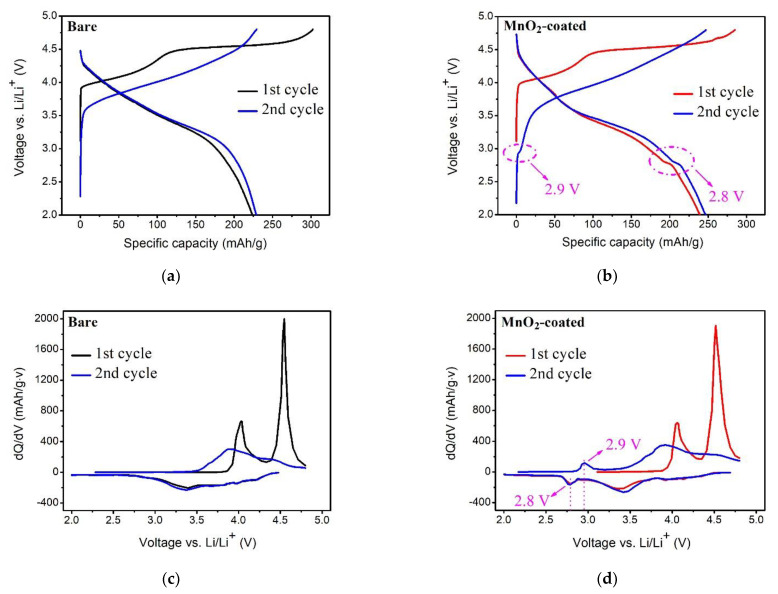
The electrochemical characteristics of the bare and MnO_2_-coated LTMO at 0.05 C. (**a**,**b**) Charge and discharge curves and (**c**,**d**) *dQ/dV* profiles.

Figure 5 displays the electrochemical characteristics of the bare and MnO_2_-coated LTMO at 0.05 C for the first two cycles. The first charge curve shows no evident change after MnO_2_ coating. Both curves have an inclined plateau at less than 4.5 V and a gentle plateau over 4.5 V (Figure 5a,b), which always appears in LTMO [3,4]. The inclined voltage plateau is derived from the variations of Ni^2+^ to Ni^4+^, as well as Co^3+^ to Co^4+^, in the LiMn_0.333_Ni_0.333_Co_0.333_O_2_ component [31]. The gentle plateau emerged only in the first charge process, reflecting the activation of the Li_2_MnO_3_ component, and was accompanied by the removal of Li_2_O [3,7,13,17]. As for the first discharge curves, the MnO_2_-coated LTMO exhibited a small potential plateau around 2.8 V, but not in the bare sample. Meanwhile, an additional corresponding charge plateau around 2.9 V occurred in the second charge curve for only the MnO_2_-coated sample.

The *dQ*/*dV* profiles for the first two cycles of the LTMO before and after MnO_2_ coating are depicted in Figure 5c,d. For the *dQ*/*dV* profiles of the bare and MnO_2_-coated LTMO during the initial charge, the anodic peak at 4.0 V was correlated to the inclined plateau in the charge curve (Figure 5a,b), and another peak at 4.5 V was associated with the gentle plateau. Moreover, a cathodic peak at 2.8 V and an anodic peak at 2.9 V appeared in the *dQ*/*dV* profiles of the MnO_2_-coated LTMO (Figure 5d), while these two peaks could not be found for the bare sample (Figure 5c), which is in keeping with the information in Figure 5a,b. According to the previous literature [44], these phenomena could stem from the insertion of Li^+^ into the coated MnO_2_ in the first discharge process and reversible extraction in the following charge process. The results prove that the MnO_2_ layer with electrochemical activity was well coated on the surface of the LTMO.

The charge and discharge capacities of the bare LTMO were 302.4 and 224.2 mAh/g, respectively (Figure 5a). In contrast, although the charge capacity of the MnO_2_-coated LTMO decreased to 285.1 mAh/g, the discharge capacity climbed up to 239.1 mAh/g (Figure 5b). Consequently, after surface coating, the irreversible capacity loss fell from 78.2 to 46.0 mAh/g, while the coulombic efficiency rose from 74.1% to 83.9%. The electrochemical redox process of the MnO_2_ coating layer could be partially responsible for the enhanced first discharge capacity and the reduced initial irreversible capacity loss. Furthermore, the growth of the SEI was reduced, which was ascribed to the repression of the side reaction between the LTMO particles and electrolyte by the MnO_2_ modification, which can be another benefit for the promoted electrochemical performance [42,43,44]_._

### 3.4. The Cyclic Performance of Bare and MnO_2_-Coated LTMO

**Figure 6:**
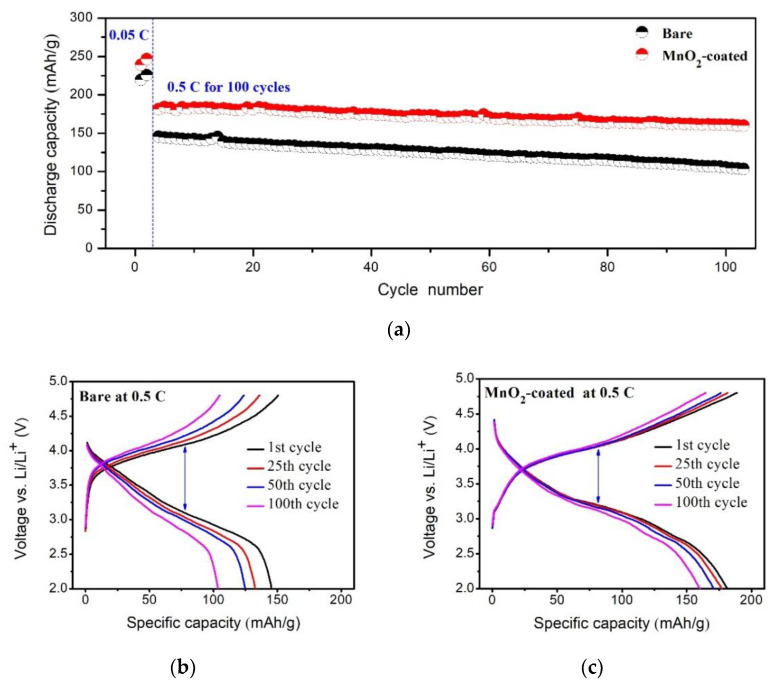
(**a**) Cyclic performance at 0.5 C. Charge and discharge curves of the (**b**) bare LTMO and (**c**) MnO_2_-coated LTMO for different cycles.

Figure 6 displays the cyclic performance of the bare and MnO_2_-coated LTMO at 0.5 C for 100 cycles after an activation process at 0.05 C for two cycles. The cyclic performance at 0.5 C of the LTMO cathode was remarkably enhanced after the MnO_2_ coating (Figure 6a). Specifically, the bare LTMO delivered 145.6 and 103.5 mAh/g for the first and 100th cycles, and the corresponding retention was 71.1%. In contrast, the MnO_2_-coated LTMO released a higher capacity of 181.5 mAh/g at the first cycle and retained 160.1 mAh/g at the end with a retention rate of 88.2%. In addition, the MnO_2_-coated sample exhibited less polarization compared to the bare one as the cycle proceeded (Figure 6b,c). Since MnO_2_ can hinder the direct contact between the LTMO particles and the electrolyte [42,43,44], the superior cyclic stability after the surface coating was due to the inhibition of electrolyte corrosion on the surface of LTMO at high potential. Similarly to the metal oxides reported in previous studies (Table 3), MnO_2_ can evidently improve the electrochemical performance of LTMO, and especially has the advantage of reducing the initial irreversible capacity loss.

### 3.5. The Rate Capabilities of Bare and MnO_2_-Coated LTMO

**Figure 7:**
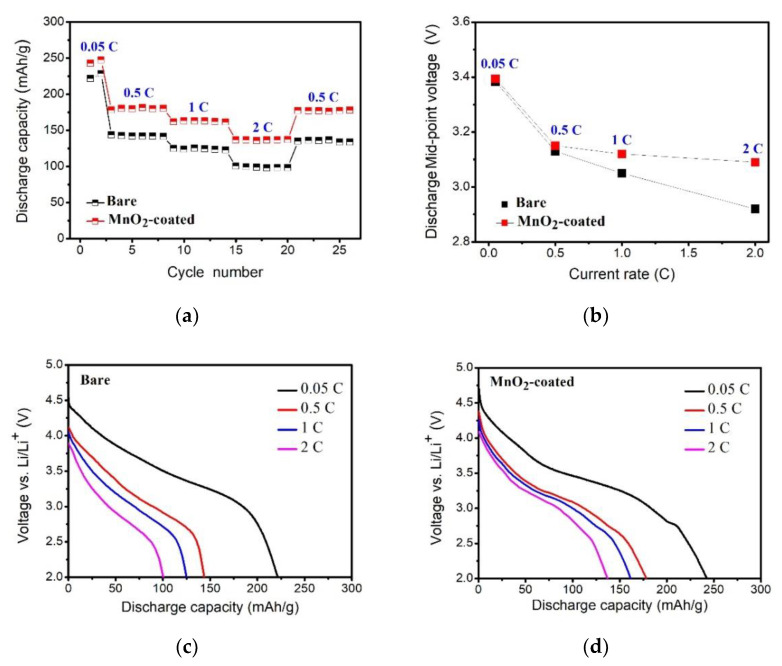
The electrochemical properties of the bare and MnO_2_-coated LTMO evaluated at various C rates. (**a**) The rate capabilities. (**b**) The discharge midpoint voltage. (**c**,**d**) Discharge curves.

The bare and MnO_2_-coated LTMO were successively tested at 0.05, 0.5, 1, 2, and 0.5 C to evaluate their rate capabilities. As exhibited in Figure 7a, the MnO_2_-coated LTMO delivered an elevated capacity in comparison with the bare sample at all C rates. Moreover, as the C rate increased, the discharge midpoint voltage difference between the MnO_2_-coated LTMO and the bare LTMO showed an upward tendency (Figure 7b), and the discharge curves of the bare sample (Figure 7c) exhibited more polarization compared to the MnO_2_-coated sample (Figure 7d). The detailed discharge capacities of the bare LTMO at 0.05, 0.5, 1, and 2 C were 221.7, 143.7, 125.0, and 100.5 mAh/g, respectively. In contrast, the MnO_2_-coated LTMO was able to deliver improved capacities of 242.8, 178.3, 161.8, and 136.9 mAh/g under identical test conditions.

### 3.6. The EIS Measurements of Bare and MnO_2_-Coated LTMO

**Figure 8:**
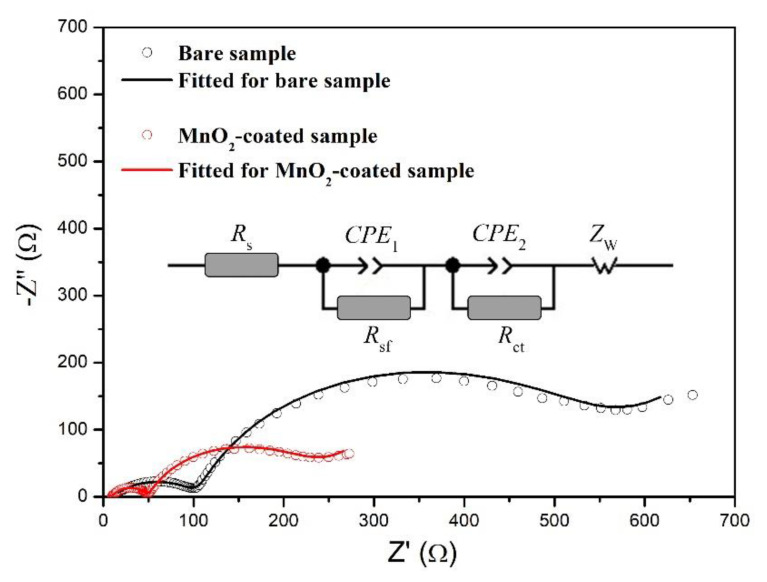
Nyquist plots of the bare and MnO_2_-coated LTMO and the equivalent circuit for fitting.

To seek the origin of the enhanced electrochemical properties, EIS experiments were conducted on the bare and MnO_2_-coated LTMO. Before the EIS tests, both the bare and MnO_2_-coated samples were charged to 4.6 V after 40 cycles at 0.5 C to achieve an identical status. Figure 8 demonstrates the obtained and fitted Nyquist plots, as well as the equivalent circuit. Similarly, the EIS spectra of the bare and MnO_2_-coated LTMO exhibited a pair of well-defined semicircles and a short, straight line that rose slowly. The intersection between the left side of the initial semicircle and the *x-*axis reflects the total ohmic resistance (*R*_s_) [45]. The initial semicircle in the high frequency reflects the impedance (*R*_sf_) for Li^+^ transmission through the interfacial film (SEI layer) of the cathode particle, and the subsequent semicircle in intermediate frequency indicates the resistance of the charge transfer reaction (*R*_ct_). The short line in the low frequency is relevant with the Warburg impedance (*Z*_w_), which reflects Li^+^ diffusion inside the cathode particles [28,46].

The fitted values of *R*_s_, *R*_sf_, and *R*_ct_ are listed in Table 4. *R*_s_, which contains the impedance of the two electrodes, separator, and electrolyte, showed close values for the two samples. Nonetheless, the values of *R*_sf_ and *R*_ct_ for the MnO_2_-coated sample were 39.7 and 167.8 Ω, exhibiting values that remarkably declined compared to those of the bare one (94.5 and 422.6 Ω). The side reaction occurring on the cathode surface with the electrolyte was significantly impaired after the MnO_2_ modification, leading to a decrease in *R*_sf_ [42]. Furthermore, the electrochemically active MnO_2_ coating layer was conducive to Li^+^ transport [43,44], together with the reduced *R*_sf_, which accounted for the decrease in *R*_ct_ for the coated sample. The EIS results demonstrate that the coated MnO_2_ was able to accelerate the transmission kinetics of Li^+^ through the interfacial film and sped up the charge transfer reaction, revealing the origin of the superior electrochemical performance of the MnO_2_-coated LTMO.

## 4. Conclusions

In summary, a Li-rich layered cathode material, LTMO, was prepared and then successfully coated with MnO_2_. Firstly, the MnO_2_ coating layer can inhibit the electrolyte from corroding the LTMO surface and can optimize the formation of SEI. Moreover, lithium ions are reversibly inserted into/extracted from MnO_2_, which affords an additional capacity. Compared with the bare LTMO, the MnO_2_-coated sample displayed a better first discharge capacity, lower initial irreversible capacity loss, superior cyclic performance, and promoted rate capability. After the MnO_2_ coating, the first discharge capacity rose from 224.2 to 239.1 mAh/g, and the initial irreversible capacity loss fell from 78.2 to 46.0 mAh/g. Furthermore, the MnO_2_-coated LTMO delivered a cyclic retention of 88.2% after 100 cycles at 0.5 C, which is more competitive than that of the bare LTMO with a value of 71.1%. At a high current density of 2 C, the discharge capacity increased from 100.5 to 136.9 mAh/g after the modification with MnO_2_. The EIS results illustrate that the electrochemically active MnO_2_ can enhance the transmission kinetics of lithium ions through the interfacial film and can speed up the charge transfer reaction. Therefore, MnO_2_ should be a beneficial surface modification material for promoting the electrochemical properties of LTMO.

## Figures and Tables

**Table 1:** Lattice parameters of the LTMO before and after the MnO_2_ modification.

Sample	*a* (Å)	*c* (Å)	*c*/*a*	*I*_(003)_/*I*_(104)_
Bare	2.8501	14.2384	4.9958	2.24
MnO_2_-coated	2.8508	14.2364	4.9938	2.26

**Table 2:** The elemental composition of the bare and MnO_2_-coated LTMO determined with ICP-AES.

Sample	Measured Atomic Ratio		
	Mn	Ni	Co
Bare	0.541	0.130	0.129
MnO_2_-coated	0.559	0.131	0.128

**Table 3:** Comparison of the data of MnO_2_ with other metal oxides reported as coating materials for LTMO.

Coating Material [References]	Initial Irreversible Capacity Loss (mAh/g)		Cyclic Retention	
	Bare Sample	Coated Sample	Bare Sample	Coated Sample
This work MnO_2_ (3 wt %)	78.2	46	85.4% (50 cycles)	94.3% (50 cycles)
			71.1% (100 cycles)	88.2% (100 cycles)
ZrO_2_ (1 wt %) [10]	~70.3	~71.9	87.5% (50 cycles)	94.9% (50 cycles)
Al_2_O_3_ (3 wt %) [23]	75	41	89.5% (50 cycles)	94% (50 cycles)
TiO_2_ (3 mol %) [25]	75.5	~60	63% (90 cycles)	87% (90 cycles)
MgO (2 wt %) [27]	74.1	73.7	70.7% (100 cycles)	96.4% (100 cycles)
ZnO (20 ALD layers) [28]	77.5	50.8	85.3% (100 cycles)	97.5% (100 cycles)
Pr_6_O_11_ (3 wt %) [29]	73.1	47.8	79.2% (50 cycles)	97.9% (50 cycles)
Er_2_O_3_ (4 wt %) [30]	64	55	84% (300 cycles)	89% (300 cycles)

**Table 4:** Lattice-fitted values of the parameters according to the equivalent circuit.

Sample	*R*_s_ (Ω)	*R*_sf_ (Ω)	*R*_ct_ (Ω)
Bare	10.3	94.5	422.6
MnO_2_-coated	9.6	39.7	167.8
